# Multidimensional expectations of radiotherapy among patients with cancer – A cross-sectional study (ExpectRT)^☆^

**DOI:** 10.1016/j.tipsro.2026.100422

**Published:** 2026-08-04

**Authors:** Alexander Fabian, Johannes A.C. Laferton, Natalie Grund, Christian Schulz, Olaf Wittenstein, Claudia Schmalz, Katrin Liethmann, Dirk Rades, David Krug

**Affiliations:** a Department of Radiation Oncology, University Hospital Schleswig-Holstein Campus Kiel, Kiel, Germany; b Institute for Mental Health and Behavioral Medicine and Department of Medicine, Health and Medical University Potsdam, Potsdam, Germany; c Department of Psychooncology, University Hospital Schleswig-Holstein Campus Kiel, Kiel, Germany; d Department of Radiation Oncology, University Hospital Schleswig-Holstein Campus Lübeck, Lübeck, Germany

**Keywords:** Radiotherapy, Treatment Expectations, Neoplasms, Patient-reported Outcome Measures, Psychological Distress, Quality of Life, Social Determinants of Health

## Abstract

**Background:**

Patients' expectations correlate with outcomes. Yet little is known about multidimensional expectations and associated factors among patients with cancer awaiting radiotherapy.

**Methods:**

We conducted a cross-sectional survey to explore expectations and associated factors based on the Likert-type Treatment Expectation Questionnaire (TEX-Q) among patients with cancer awaiting radiotherapy. The TEX-Q mean (higher score = more positive overall expectations) was the primary outcome. Secondary outcomes included the TEX-Q Treatment benefit (higher score = more beneficial expectations) and Adverse events (higher score = worse adverse events expectations) subscales. TEX-Q scores range from 0 to 10. We performed univariate and multivariate analyses (MVA).

**Results:**

Among 368 eligible patients, 140 participated and 133 were analysable. The median age was 67.5 years (interquartile range: 18.8) and the female-male ratio was 1:1. The TEX-Q mean value was 7.0 (SD:1.8), the Treatment benefit mean was 8.6 (SD:1.7), and the Adverse events mean was 4.3 (SD:2.3). Treatment benefit and Adverse events expectations did not correlate. On MVA, overall expectations were worse among patients living alone (*p* = 0.01), with higher education (*p* < 0.001), and receiving concomitant chemotherapy (*p* = 0.048). Treatment benefit expectations were worse among patients with higher education (*p* = 0.03) and with palliative/unknown intent (*p* = 0.003). Adverse event expectations were more pronounced among patients living alone (*p* = 0.01), inpatients (*p* = 0.04) and more distressed patients (p = 0.01).

**Conclusions:**

Patients' expectations were favourable overall. Patients living alone, treated with palliative intent and at more distress expected worse outcomes which may require adapted communication strategies or supportive measures. Future longitudinal studies should investigate expectation-outcome pairings and interventions to realistically improve patients' expectations regarding radiotherapy.

## Highlights

• Multidimensional assessment of patient's radiotherapy expectations.
• Treatment benefit and adverse events expectations unrelated.
• Higher education and palliative intent determined lower benefit expectations.
• Living alone and higher distress determined higher adverse event expectations.
• Previous positive experiences of RT determined favourable expectations.

## Introduction

Patients' expectations are future-directed beliefs from the patient's view on how a health intervention may benefit or harm his or her life [1]. Positive or negative patients' expectations correlate with actual beneficial or adverse outcomes, known as the placebo or nocebo effect, respectively [2]. For example, a prospective cohort study of patients with breast cancer treated with endocrine therapy showed that negative pre-treatment expectations correlated with worse side-effects and quality of life (QoL) at longitudinal follow-up [3]. Although causal relationships of the expectation-outcome pairing require further understanding, the patient-clinician communication plays a major role in patients' expectations [2], [4], [5]. However, the lack of adequate measures of patients' expectations has long been a barrier to a deeper understanding of patients' expectations per se and their associations with outcomes. In fact, research on patients' expectations often relied on unidimensional, study specific, and often modestly validated questionnaires without theoretical grounding [1], [6]. To bridge this gap, the Treatment Expectation Questionnaire (TEX-Q) has recently been developed [7]. The TEX-Q is a generic, multidimensional, and theory-based questionnaire on treatment expectations from a patient's view that has undergone validation which involved patients with cancer [7].

Radiotherapy forms an essential pillar in the treatment of patients with cancer, as approximately 50% have an indication for radiotherapy [8]. Radiotherapy increases survival, offers palliation and has undergone remarkable advances in the past decades [9], [10], [11]. Yet radiotherapy received increasingly negative lay media coverage [12]. In response, refined wordings in the field of radiotherapy have been proposed such as the use of “side-effects” instead of “toxicity” during patient-clinician communication [13]. In fact, the framing of benefits and harms may impact treatment expectations and potentially outcomes [2].

Although deterministic tissue response prevails [11], the context of radiotherapy appears as a prime case study to investigate patients' expectations. The term “radiation” is often loaded with negative associations and there is usually no discernable immediate action-outcome link after radiotherapy when compared to surgery. These facts might facilitate misconceptions. Misconceptions of radiotherapy have been shown to associate with anxiety and radiotherapy refusal among patients with cancer [14]. Previous studies on treatment expectations of radiotherapy mainly relied on study specific questionnaires [15], [16], [17], [18], [19], [20]. Furthermore, most previous studies applied unidimensional expectation assessments (e.g. only benefit expectations) or focused on patients' expectations regarding prognosis and treatment intent [21], [22], [23]. Hence, we lack a deeper understanding of patients' expectations over a broad cohort of patients with cancer planned for radiotherapy on the basis of a validated multidimensional tool. Therefore, we surveyed patients with various types of cancer planned for radiotherapy using the TEX-Q. We aimed at describing the distribution of expectations prior to radiotherapy and at investigating potentially associated factors. This data could inform current practice and support the planning of future expectations research.

## Methods and materials

### Study design

We conducted a protocol-based cross-sectional survey at a single academic cancer centre to explore expectations of radiotherapy among patients with cancer. The study was approved by the local ethics committee (D 581/25). Patients were eligible if (i) they had a cancer diagnosis and had given informed consent for radiotherapy, (ii) were able to understand and complete questionnaires in German, and (iii) were aged 18 years or older.

Patient recruitment took place at the planning-CT. Hence, participating patients had already undergone radiotherapy consent and radiotherapy had not started ensuring a representative stage of the patient journey. Patients may have given informed consent for radiotherapy by one of eight different physicians. From 11/2025 to 02/2026, radiographers invited all potentially eligible patients on-site at the day of the planning-CT offering a study information sheet, two paper-based questionnaires, and a sealing envelop. Patients willing to participate returned the completed questionnaires in the sealed envelope which was then put in to a locked box ensuring anonymous data capture. Hence, given informed consent for study participation applied after patients had acknowledged the patient information sheet and voluntarily returned the questionnaires. There was no signed informed consent sheet in light of the anonymous study design which was chosen pragmatically to facilitate recruitment. We estimated the study participation rate by screening the planning-CT schedule for appointments and subtracting obviously ineligible patients (e.g., brachytherapy planning-CT). Hence, our reported values are a conservative estimation of the participation rate as less patients might have been eligible e.g. due to unobvious language barriers. We respected the STROBE guideline for reporting as applicable [24].

### Variables and outcomes

All data were patient-reported. We used two questionnaires for data sampling: a covariable questionnaire and the TEX-Q [7].

The covariable questionnaire was pretested on and approved by two potentially eligible patients. It contained general patient characteristics (gender, age, cohabitation status, education in years of school) and disease characteristics (tumor entity, intent of radiotherapy, use of concomitant chemotherapy, hospitalization). Furthermore, it contained single items from validated patient-reported outcomes measures. First, this included two questions from the Patient Satisfaction with Cancer Care Questionnaire (PSCC) assessing the level of shared-decision making (PSCC question 3, “I felt included in decisions about my health.”) and the level of received needed advice (PSCC question 11, “I was able to get the advice I needed about my health issues”) in the context of radiotherapy [25]. The PSCC is rated on a 5-point Likert scale with higher values indicating higher agreement. Second, we included the National Comprehensive Cancer Network (NCCN) distress thermometer rated on an 11-point scale with higher values indicating higher distress [26]. Lastly, we included questions 29 & 30 of the European Organisation for Research and Treatment of Cancer (EORTC) QLQ-C30 to capture the global health status/ QoL. Both questions are rated on a 7-point scale and merged in analysis for interpretation of the global health status/ QoL in which higher values indicate better global health status/ QoL [27].

The TEX-Q is a theory-based and validated questionnaire on patients' treatment expectations [7]. The TEX-Q covers various aspects of expectations: (i) expectations on treatment benefit, (ii) positive impact, (iii) expectations on adverse effects, (iv) negative impact, (v) process expectations, and (vi) behavioral control expectations. These various aspects of expectations are hence covered by six subscales, assessed by 15 items in total and on an 11-point rating scale per item. All 15 items are expressed in the TEX-Q mean score (i.e., a”sum score”) in which higher values indicate more positive expectations. In addition, the TEX-Q assesses if patients previously received the planned treatment. If so, patients are asked additional 11-scaled questions on the previous overall experience and improvements after the previous therapy with higher scores indicating more beneficial experiences. Our primary outcome was the TEX-Q mean score to investigate patients' expectations prior to radiotherapy. Furthermore, we assessed the TEX-Q subscales Treatment benefit and Adverse events separately as secondary outcomes, as these subscales appeared unrelated in the validation study [7].

### Statistical analysis

All analyses were exploratory given the lack of previous data. We used descriptive statistics to display cohort characteristics. Item scores of the PSCC, NCCN distress thermometer, and EORTC QLQ-C30 questionnaires were calculated based on respective primary publications [25], [26], [27]. The TEX-Q mean was calculated based on all 15 items after inverting item 7–11 [7]. The TEX-Q subscales Treatment benefit and Adverse events were calculated from item 1–3 and 7–9, respectively. We used Cronbach's ⍺, Skewness, and Kurtosis to display in-sample psychometric properties of the TEX-Q. We used one-way analysis of variance (ANOVA), Spearman, and Pearson correlation to investigate univariate associations of covariables with TEX-Q scores. As predefined for multivariate analysis, we used multiple regression models to investigate factors associated with TEX-Q mean, Treatment benefit subscale, and Adverse event subscale scores. For each TEX-Q score we performed a full model and a model with selected variables based on backwards elimination [28]. We used 1000 bootstrap samples to assess the stability of selected variables. Concerning missing data, only patients with at least 80% of completed TEX-Q questions (items 1–15) were included for analysis as it was the case in the TEX-Q validation study [7]. We used multiple imputation by chained equations (imputations *n* = 5; iterations *n* = 10) for patients that were included in the analysis but had otherwise randomly missing covariables or TEX-Q data. We predefined a minimum of 100 participating patients in the set recruitment period. We considered a two-sided *p*-value <0.05 as statistically significant. Given the exploratory design of our study, we did not adjust for multiple testing [29]. Analyses were performed using JASP v0.19.3 (JASP Team [2025], Amsterdam, The Netherlands), IBM SPSS Statistics v31.0 (IBM Corp. [2025], Armonk, NY, USA), and R 4.6.0 (R Core Team, 2026).

## Results

### Recruitment and cohort characteristics

**Fig. 1:**
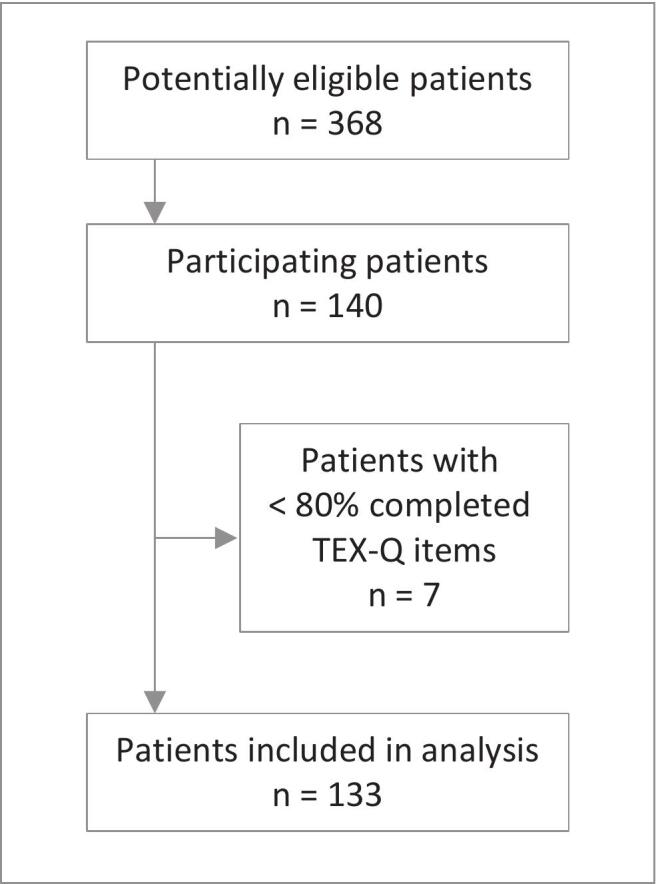
Study flow chart. Abbreviations: TEX-Q, Treatment Expectations Questionnaire.

Of 368 potentially eligible patients, 140 patients participated resulting in a participation rate of 38% (Fig. 1). The analysed data set contains 133 patients as seven patients completed <80% of the TEX-Q. Concerning characteristics of analysed patients, 49.6% were female, the median age was 67.5 years, and the most common tumor entities were breast cancer and prostate cancer, respectively (Table 1). Further, 83.5% reported to be treated with curative intent and 23.3% had a previous course of radiotherapy. Among the latter, the overall experience and improvements after previous radiotherapy were rated at a mean of 7.3 (standard deviation[SD]:2.3) and 6.7 (SD:3.0), respectively. In the whole analysed data set, the mean distress score was 5.3 (SD:2.6) and the mean global health status/QoL score was 58.3 (SD:21.2), respectively.

**Table 1.** Characteristics of 133 analysed patients. Absolute numbers are given in brackets. Numbers may not add up to 100% due to rounding error. Abbreviations: IQR, interquartile range; QoL, quality of life; SD, standard deviation.

		Participants 100% (133)
Sex	Female: male	49.6%: 50.4% (66: 67)
	Missing	0
Age	Years	Median: 67.5; IQR: 18.8
	Missing	11.3% (15)
Cohabitation status	Living alone	22.6% (30)
	Living with partner	75.2% (100)
	Missing	2.3% (3)
Education	< 10 years of school	27.1% (36)
	10 years of school	33.1% (44)
	> 10 years of school	37.6% (50)
	Missing	2.3% (3)
Tumor entity	Breast	27.1% (36)
	Prostate	24.8% (33)
	Brain	7.5% (10)
	Head and neck	6.0% (8)
	Lung	5.3% (7)
	Bone Metastasis	5.3% (7)
	Gynaecological	3.8% (5)
	Lymphoma	3.8% (5)
	Other	16.5% (22)
	Missing	0
Indication of radiotherapy	Curative	83.5% (111)
	Palliative	9.8% (13)
	“Not sure”	6.0% (8)
	Missing	0.8% (1)
Concomitant chemotherapy	Yes	25.6% (34)
	No	72.9% (97)
	Missing	1.5% (2)
Hospitalized during radiotherapy	Yes	18.8% (25)
	No	78.9% (105)
	Missing	2.3% (3)
Previous radiotherapy	Yes	23.3% (31)
	No	76.7% (102)
	Missing	0
Level of shared decision-making	PSCC	Median: 4; IQR: 1
	Missing	0
Level of received needed advice	PSCC	Median: 5; IQR: 1
	Missing	0
Distress	NCCN thermometer	Mean: 5.3; SD: 2.6
	Missing	0
Global health status/ QoL	EORTC QLQ-C30	Mean: 58.3; SD: 21.2
	Missing	1% (2)

### TEX-Q expectations results

Table 2 displays single item, subscale, and TEX-Q mean results of the TEX-Q in the analysed data set including in-sample psychometric properties. Of note, Cronbach's ⍺ ranged from 0.71 to 0.93 suggesting acceptable to excellent reliability of subscales as well as the TEX-Q mean. The positively valenced expectation scales, particularly TEX-Q Treatment benefit and TEX-Q Positive impact as well as the TEX-Q mean score, were negatively skewed, indicating generally high positive treatment expectations and some ceiling tendency in this sample.

**Table 2.** Treatment Expectation Questionnaire (TEX-Q) responses and in-sample psychometric item as well as scale characteristics among 133 analysed patients. Subscales and TEX-Q mean (=sum score) results are shown in bold font. The TEX-Q mean score is calculated as mean of all items inverting item 7–11. Abbreviation: SE, standard error; SD, standard deviation.

Subscale	Items	Mean (SD)	Cron- bach's ⍺	Skewness (SE)	Kurtosis (SE)	Missing *% (n)*
**Treatment benefit** (Higher score = greater benefit)		**8.6 (1.7)**	**0.71**	**−1.55 (0.22)**	**2.18 (0.43)**	**5% (7)**
	1. How much relief of your symptoms do you expect from the treatment?	8.2 (2.8)		−1.87 (0.22)	2.57 (0.43)	5% (7)
	2. How much benefit do you expect from the treatment?	9.1 (1.5)		−2.11 (0.21)	4.78 (0.42)	0
	3. How much do you expect your health will improve as a result of the treatment?	8.6 (2.0)		−2.16 (0.21)	5.31 (0.42)	0

**Positive impact** (Higher score = greater positive impact)		**7.6 (2.9)**	**0.93**	**−1.34 (0.22)**	**0.8 (0.43)**	**6% (8)**
	4. How much improvement do you expect in your ability to do your daily activities?	7.5 (3.3)		−1.39 (0.21)	0.69 (0.42)	3.8% (5)
	5. How much do you expect the treatment will improve your quality of life?	7.8 (2.9)		−1.63 (0.21)	1.88 (0.42)	1.5% (2)
	6. How much improvement do you expect in your ability to fulfil your day-to-day responsibilities?	7.4 (3.2)		−1.30 (0.21)	0.50 (0.42)	3.0% (4)

**Adverse events** (Higher score = worse adverse events)		**4.3 (2.3)**	**0.89**	**0.16 (0.21)**	**−0.53 (0.42)**	**0**
	7. To what extent do you expect risks from the treatment?	3.9 (2.6)		0.22 (0.21)	−0.66 (0.42)	0
	8. How much distress do you expect the treatment will cause?	4.7 (2.6)		0.03 (0.21)	−0.79 (0.42)	0
	9. To what extent do you expect side effects or other unwanted effects from the treatment?	4.4 (2.5)		0.24 (0.21)	−0.69 (0.42)	0

**Negative impact** (Higher score = worse negative impact)		**3.8 (2.4)**	**0.83**	**0.10 (0.21)**	**−0.68 (0.42)**	**1.5% (2)**
	10. How much do you expect the treatment will reduce your quality of life?	4.0 (2.8)		0.30 (0.21)	−0.71 (0.42)	0
	11. How much do you expect the treatment will limit your day-to-day responsibilities?	3.7 (2.5)		0.07 (0.21)	−0.99 (0.42)	1.5% (2)

**Process** (Higher score = greater approval)		**7.6 (1.9)**	**0.73**	**−0.93 (0.21)**	**1.0 (0.42)**	**0.8% (1)**
	12. To what extent do you expect the treatment procedure or process to be straight-forward?	6.8 (2.5)		−0.76 (0.21)	0.05 (0.42)	0.8% (1)
	13. To what extent do you expect to be satisfied with the treatment procedure or process?	8.4 (1.7)		−1.31 (0.21)	2.12 (0.42)	0

**Behavioral control** (Higher score = greater approval)		**6.6 (2.9)**	**0.90**	**−0.70 (0.21)**	**−0.42 (0.42)**	**1.5% (2)**
	14. To what extent do you expect to be responsible for the success of the treatment?	6.9 (3.0)		−0.83 (0.21)	−0.32 (0.42)	1.5% (2)
	15. To what extent do you expect your own behaviour to influence the success of the treatment?	6.2 (3.0)		−0.53 (0.21)	−0.70 (0.42)	0.8% (1)

**TEX-Q mean** (Higher score = more positive expectations)		**7.0 (1.8)**	**0.87**	**−1.07 (0.22)**	**2.17 (0.44)**	**9% (12)**

**Fig. 2:**
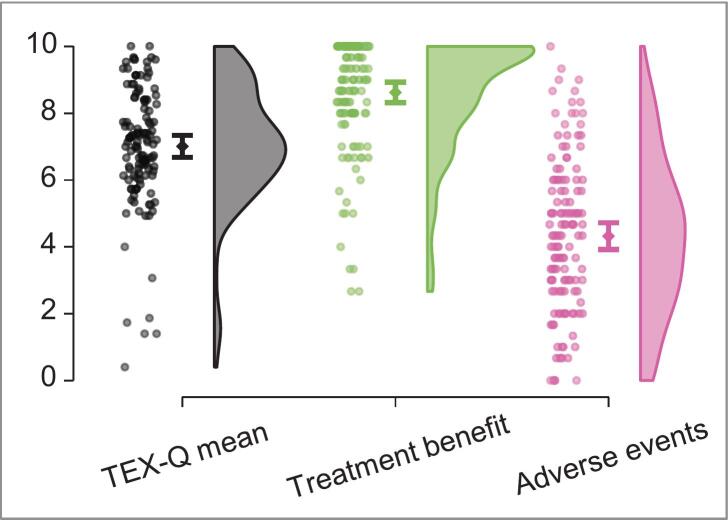
Raincloud plots of Treatment Expectation Questionnaire (TEX-Q) mean (= sum score) values (grey colour), Treatment benefit subscale values (green colour) and Adverse events subscale values (light purple colour) among patients of the analysed data set (*n* = 133). Plots show data points, means with 95% confidence intervals, and data densities for each scale, respectively. Higher values of the TEX-Q mean relate to more positive overall expectations. Higher values of the Treatment benefit subscale relate to greater benefit expectations. Higher values of the Adverse events subscale relate to worse adverse events expectations. (For interpretation of the references to colour in this figure legend, the reader is referred to the web version of this article.)

Patients scored a TEX-Q mean value of 7.0 (SD:1.8), a Treatment benefit subscale mean value of 8.6 (SD:1.7), and an Adverse events subscale mean value of 4.3 (SD:2.3) (Fig. 2). There was no significant correlation of the TEX-Q Treatment benefit with the Adverse events subscale (Pearson's r, −0.15 [95% Confidence Interval(CI)], −0.31–0.03]; *p* = 0.1). Hence, patients who expected more treatment benefit from radiotherapy did not necessarily expect less adverse events and vice versa. This supported the rationale for a parallel evaluation of the TEX-Q mean score along with the Treatment benefit and Adverse event subscale.

### Univariate associations of covariables with expectations

Next, we assessed univariate associations of covariables with the TEX-Q mean score, the Treatment benefit subscale, and the Adverse events subscale, respectively. Concerning nominal covariables, patients living alone reported significantly lower TEX-Q mean scores and higher Adverse events subscale scores as compared to patients who lived with a partner (Table 3). This means that patients who lived alone had overall more negative expectations towards radiotherapy and expected more adverse events. Further, patients who reported to be treated with palliative intent or were unsure of the treatment intent had significantly lower TEX-Q Treatment benefit subscale mean values as compared to patients treated with curative intent. In addition, inpatients reported significantly higher TEX-Q Adverse events scores as compared to outpatients. Neither sex, nor tumor entity, nor concomitant chemotherapy, nor previous radiotherapy were associated with TEX-Q scores.

**Table 3.** Associations of Treatment Expectation Questionnaire (TEX-Q) mean values, Treatment benefit subscale values and Adverse events subscale values with categorical independent variables in the analysed data set (n = 133) as per one-way ANOVA. Abbreviation: SD, standard deviation.

	TEX-Q mean		TEX-Q Treatment benefit subscale		TEX-Q Adverse events subscale	
**Variable**	M (SD)	p	M (SD)	p	M (SD)	p

**Sex**		0.8		0.8		0.8
Female	7.1 (1.7)		8.6 (1.9)		4.3 (2.4)	
Male	7.0 (7.0)		8.6 (1.5)		4.4 (2.2)	

**Cohabitation status**		**0.008**		0.5		**0.008**
Living alone	6.4 (1.5)		8.4 (1.8)		5.3 (2.2)	
Living partner	7.3 (1.5)		8.7 (1.7)		4.0 (2.3)	

**Tumor entity**		0.6		0.2		0.06
Breast	7.5 (1.3)		8.8 (1.3)		4.1 (2.1)	
Prostate	7.1 (1.6)		8.8 (1.6)		4.1 (2.5)	
Brain	6.9 (2.2)		8.1 (2.7)		3.2 (2.5)	
Head and neck	7.4 (1.8)		9.4 (0.8)		5.9 (3.3)	
Lung	7.1 (1.2)		8.4 (1.2)		4.4 (1.6)	
Bone Metastasis	6.6 (1.4)		7.1 (2.3)		3.3 (1.5)	
Gynaecological	7.2 (1.3)		8.8 (1.8)		4.4 (1.4)	
Lymphoma	7.4 (0.8)		9.3 (1.0)		3.3 (1.4)	
Other	6.5 (1.9)		8.3 (2.1)		5.5 (2.2)	

**Indication**		0.3		**0.005**		0.4
Curative	7.1 (1.6)		8.8 (1.6)		4.4 (2.4)	
Palliative/ ”not sure”	6.8 (1.6)		7.7 (2.2)		3.9 (1.9)	

**Concomitant chemotherapy**		0.1		0.2		0.07
Yes	6.7 (1.9)		8.3 (2.0)		4.9 (2.6)	
No	7.2 (1.4)		8.7 (1.6)		4.1 (2.2)	

**Hospitalized during radiotherapy**		0.2		0.5		**0.01**
Yes	6.7 (1.9)		8.4 (2.2)		5.4 (2.9)	
No	7.2 (1.5)		8.7 (1.6		4.1 (2.1)	

**Previous radiotherapy**		0.9		0.9		0.5
Yes	7.1 (1.4)		8.6 (1.9)		4.0 (1.9)	
No	7.1 (1.6)		8.6 (1.7)		4.4 (2.5)	

Concerning ordinal variables, patients with higher education levels reported significantly lower TEX-Q mean values as well as lower TEX-Q Treatment benefit subscale values (Supplementary Table 1). Neither levels of shared decision-making nor levels of received needed advice were associated with any TEX-Q score.

Concerning continuous variables, higher distress was significantly associated with higher TEX-Q Adverse events subscale values. Within the subset of patients who reported to have had a previous course of radiotherapy (*n* = 31), greater subjective improvements after the previous radiotherapy were significantly associated with higher TEX-Q mean scores (Supplementary Table 2). Neither age nor global health status/ QoL were associated with any TEX-Q score.

### Multivariate associations of covariables with expectations

Next, we assessed multivariate associations of covariables with the TEX-Q mean score, the Treatment benefit subscale, and the Adverse events subscale. As prespecified, we conducted a full model analysis and then a backward elimination analysis (“final models”) for all three analysed TEX-Q scores. Model parameters as well as model assumption checks are reported in Supplementary Table 3 and Supplementary Table 4. Variable selection stability of the final models is shown in Supplementary Table 5. Adjusted R^2^ values ranged from 0.04 to 0.09 for full models and from 0.08 to 0.13 for final models, respectively, indicating a small to moderate explanation of variance by available covariables. In the full models, TEX-Q mean scores were significantly associated with cohabitation status (B, −0.71 [95% CI], −1.37 to −0.05]; *p* = 0.04) and education (B, −0.60 [95% CI], −0.94 to −0.26]; *p* < 0.001) meaning that patients who lived alone and had higher education reported overall more negative expectations (Table 4). The TEX-Q Treatment benefit subscale was significantly associated with education (B, −0.50 [95% CI], −0.98 to −0.12]; *p* = 0.01) and indication of radiotherapy (B, 1.08 [95% CI], 0.20–1.90]; *p* = 0.02). Hence, patients with higher education levels and palliative/unsure treatment indication reported lower treatment benefit expectations. Finally, the TEX-Q Adverse event subscale was significantly associated with cohabitation status (B, 1.22 [95% CI], 0.24–2.20]; p = 0.02) and distress (B, 0.21 [95% CI], 0.04–0.38]; p = 0.01), i.e. patients who lived alone and had higher distress reported worse expectations of adverse events.

**Table 4.** Multiple regression model (“full model”) of the Treatment Expectation Questionnaire (TEX-Q) mean, Treatment benefit subscale, and Adverse events subscale as separate dependent variables in the analysed data set (n = 133). Abbreviations: CI, confidence interval; PSCC, Patient Satisfaction with Cancer Care Questionnaire; SDM, shared decision-making.

	TEX-Q mean				TEX-Q Treatment benefit subscale				TEX-Q Adverse events subscale			
Variable	B	Lower 95%-CI	Upper 95%-CI	p	B	Lower 95%-CI	Upper 95%-CI	p	B	Lower 95%-CI	Upper 95%-CI	p
Sex (Female)	0.13	−0.42	0.68	0.64	0.05	−0.56	0.67	0.86	−0.05	−0.87	0.77	0.90
Age	0.00	−0.03	0.02	0.71	0.00	−0.02	0.03	0.87	0.01	−0.03	0.04	0.69
Cohabitation status (Living alone)	−0.71	−1.37	−0.05	**0.04**	0.03	−0.71	0.77	0.94	1.22	0.24	2.20	**0.02**
Education	−0.60	−0.94	−0.26	**< 0.001**	−0.50	−0.88	−0.12	**0.01**	0.21	−0.29	0.72	0.40
Indication of radiotherapy (Curative)	0.12	−0.66	0.90	0.76	1.08	0.20	1.90	**0.02**	0.94	−0.22	2.10	0.11
Concomitant Chemotherapy (Yes)	−0.37	−1.07	0.33	0.29	−0.34	1.13	0.45	0.39	0.28	−0.76	1.32	0.60
Hospitalized (Yes)	−0.33	−1.10	0.44	0.40	−0.22	1.09	0.64	0.61	0.92	−0.23	2.06	0.12
Previous radiotherapy (Yes)	0.03	−0.63	0.69	0.93	0.28	−0.46	1.10	0.46	0.01	−0.97	1.00	0.98
Level of SDM [PSCC]	0.06	−0.33	0.45	0.75	0.15	−0.28	0.59	0.48	0.18	−0.40	0.75	0.55
Level of needed advice received [PSCC]	0.03	−0.44	0.50	0.90	0.00	−0.53	0.53	1.00	−0.20	−0.91	0.50	0.57
Distress [NCCN thermometer]	−0.03	−0.15	0.08	0.54	0.07	−0.05	0.20	0.26	0.21	0.04	0.38	**0.01**
Global health status/ QoL [EORTC QLQ-C30]	0.01	−0.01	0.02	0.49	0.01	−0.01	0.03	0.24	0.01	−0.01	0.03	0.53

The final models confirmed the significantly associated covariables found in the full models (Table 5). In addition, TEX-Q mean score was also significantly associated with concurrent chemotherapy (B, −0.58 [95% CI], −1.16 to −0.005]; *p* = 0.048) meaning that patients who received concurrent chemotherapy reported overall more negative expectations. Further, the TEX-Q Adverse event subscale was additionally associated with hospitalization (B, 1.01 [95% CI], 0.03–1.98]; p = 0.04), i.e. inpatients expected worse adverse events from radiotherapy.

**Table 5.** Multiple regression models (“final model”, based on backward elimination) of the Treatment Expectation Questionnaire (TEX-Q) mean, Treatment benefit subscale, and Adverse events subscale as separate dependent variables in the analysed data set (n = 133). Abbreviations: CI, confidence interval; SDM, shared decision-making.

	TEX-Q mean				TEX-Q Treatment benefit subscale				TEX-Q Adverse events subscale			
Variable	B	Lower 95%-CI	Upper 95%-CI	p	B	Lower 95%-CI	Upper 95%-CI	p	B	Lower 95%-CI	Upper 95%-CI	p
Cohabitation status (Living alone)	−0.76	−1.37	−0.16	**0.01**					1.27	0.36	2.19	**0.01**
Education	−0.54	−0.86	−0.22	**< 0.001**	−0.40	−0.75	−0.05	**0.03**				
Indication of radiotherapy (Curative)					1.2	0.40	1.92	**0.003**	0.98	−0.05	2.01	0.06
Concomitant chemotherapy (Yes)	−0.58	−1.16	−0.005	**0.048**								
Hospitalized (Yes)									1.01	0.03	1.98	**0.04**
Distress [NCCN]									0.20	0.05	0.34	**0.01**

## Discussion

To our knowledge, we report the first study on expectations of patients with cancer towards radiotherapy using a validated multidimensional tool, i.e. the TEX-Q [7]. Overall expectations towards radiotherapy were favourable. Treatment benefit expectations and adverse events expectations were unrelated, highlighting the need for a nuanced assessment of different dimensions of expectations. Factors associated with expectations included the cohabitation status, education level, indication of radiotherapy, concomitant chemotherapy, inpatient treatment, and distress. Each of these factors showed specific associations with overall, treatment benefit, or adverse events expectations, respectively. In the subset of patients with prior radiotherapy experience, more positive previous experiences were associated with more favourable expectations towards the current treatment.

Patients' treatment expectations appeared positive in our cohort based on the distribution of the TEX-Q mean score, Treatment benefit subscale and Adverse events subscale. External comparison, however, is difficult in light of lacking previous TEX-Q data for patients treated with radiotherapy. The confirmatory sample of the TEX-Q validation study included patients with various tumor entities and various types of treatments [7]. These patients reported a TEX-Q mean value of 7.5 (SD:1.5), a Treatment benefit subscale mean value of 8.8 (SD:1.7), and an Adverse events subscale mean value of 4.3 (SD:2.8). Although these distributions of expectations appear comparable to our cohort, comparisons should only be made with caution. An important factor that could have affected our results is the survey timing. To ensure consistency, we have chosen the day of the planning-CT scan. However, surveying patients prior to the first consultation with a radiation oncologist could have resulted in different expectations. For example, Mitera and colleagues surveyed patients prior to and after the first consultation with a radiation oncologist [20]. After the consultation, patients were more confident in radiotherapy benefits and less concerned of side-effects.

Different expectations were associated with various factors in our cohort. First, patients living alone reported worse overall expectations and worse adverse event expectations. While we are unaware of studies specifically examining the impact of cohabitation status on treatment expectations, there is some related evidence suggesting that social resources may play a role: for example, in cardiac surgery patients, higher perceived social support prior to surgery has been associated with more favourable treatment expectations [30]. Furthermore, living alone is a risk factor for worse treatment adherence and worse outcomes among patients with cancer. For example, patients living alone appear to receive less often intensified chemotherapy and less adjuvant chemotherapy [31], [32]. Further, mortality is higher among patients living alone [33]. Concerning our results one could hypothesize that patients living alone could be deprived of coping resources and hence have worse expectations towards adverse events. Regardless of the causal relations, this finding is important as already 23% of the patients in our cohort lived alone and as the prevalence of loneliness is expected to rise with aging populations [34]. Second, patients with higher education had worse overall expectations and worse treatment benefit expectations. This is a novel finding which initially appears contradictory. For example, a US-American database study reported that patients with lung cancer and higher education levels were more likely to accept treatment [35]. Causal interplays remain elusive, but it could be argued that patients with higher education levels exhibit higher levels of health literacy enabling a more realistic and pragmatic decision-making for healthcare decisions [36], [37]. Third, patients who reported to be treated with palliative/unsure intent expected less treatment benefit. Contrarily, studies have shown that patients treated with palliative intent largely overestimate the effectiveness of their treatment [23], [38], [39]. Potentially, most patients may overestimate treatment benefits and patients treated with palliative intent may do so to a lesser degree as compared to patients treated with curative intent in our cohort. However, this is speculative and requires further exploration. Lastly, more distressed patients expected worse adverse events. This association is in line with literature from other fields. A pooled analysis of six placebo-controlled trials found that higher perceived stress correlated with worse side-effect expectations [40]. Furthermore, a systematic review on side-effect expectations of medical interventions also described positive correlations with anxiety and distress [41].

Our results serve as baseline understanding for current practice and future research. This could include, first, a multicentre validation of our findings. Second, the investigation of potential expectations-outcome pairings for which a study by our group is underway. Third, interventional studies aiming to shape positive expectations on a realistic basis to potentially improve outcomes [5], [42], [43]. Such interventional trials have shown promising results in improving outcomes following cardiac or abdominal surgery [44], [45]. Similar interventional trials are underway in other fields including orthopaedic surgery, depression, or psoriasis [46], [47], [48].

Limitations of our present study include the anonymous data sampling. As all data were patient-reported, there may be imprecision in some reporting, for example concerning the indication of radiotherapy or the tumor entity. This was one of the reasons, next to the prevalent “other” entity cases, why tumor entity was not used as covariable in the multivariate analysis. Further, while the TEX-Q has been validated in patients with cancer, the validation sample was not specific to patients undergoing radiotherapy. Moreover, the conservatively estimated participation rate was modest which may have favoured selection bias. Yet cohort characteristics were well in line with previous radiotherapy survey studies [49], [50]. Finally, methodological aspects could be considered including the single-centre and cross-sectional design as well as potential instability in variable selections of our final modes given the moderate sample size.

## Conclusion

Most patients reported favourable overall radiotherapy expectations. The multidimensional assessment of expectations was essential as treatment benefit and adverse events expectations were unrelated. Patients living alone, treated with palliative intent, and at higher distress reported more negative expectations, particularly concerning adverse events. Additional efforts in communication and supportive measures may be required for these patient groups. Future studies should validate these findings, investigate longitudinal expectations-outcomes pairings, and ultimately assess interventions to realistically optimize patients' expectations which could improve outcomes.

## CRediT authorship contribution statement

**Alexander Fabian:** Writing – review & editing, Writing – original draft, Project administration, Methodology, Investigation, Formal analysis, Data curation, Conceptualization. **Johannes A.C. Laferton:** Writing – review & editing, Methodology. **Natalie Grund:** Writing – review & editing, Investigation. **Christian Schulz:** Writing – review & editing. **Olaf Wittenstein:** Writing – review & editing. **Claudia Schmalz:** Writing – review & editing. **Katrin Liethmann:** Writing – review & editing. **Dirk Rades:** Writing – review & editing, Supervision. **David Krug:** Writing – review & editing, Supervision.

## Ethics approval and consent to participate

The study was approved by the local ethics committee (Ethics Committee of the Faculty of Medicine of Kiel University; D 581/25). All patients voluntarily consented to participate.

## Declaration of Competing Interest

The authors declare the following financial interests/personal relationships which may be considered as potential competing interests: [AF received honoraria from Merck Sharp and Dohme as well as a research grant from Lilly Germany foundation outside the field of this work. ClS has received honoraria from Merck, Spectrum Therapeutics, Avextra-Pharm, Grünenthal and has received institutional research funding from Stiftung Deutsche Krebshilfe. CrS has received honoraria from Astra Zeneca. DK has received honoraria from Astra Zeneca, best practice onkologie, ESO, ESMO, Gilead, med update, Merck Sharp & Dohme, Novartis, onkowissen, Pfizer and PINK gegen Brustkrebs, served on advisory boards for Gilead and has received institutional research funding from Stiftung Deutsche Krebshilfe and Merck KGaA. DR has received research grants paid to his institution and not related to this study from the European Regional Development Fund (through the Interreg Deutschland-Danmark program) and from Merck Sharp & Dohme. JL received a speaking honorarium from Dansk Sundhedssikring A/S for a webinar for Danish healthcare providers on the application of expectation-focused communication in clinical practice. OW has received honoraria from Novocure and Brainlab AG. All other authors declare that they have no conflicts of interest to report.].

## Appendix A Supplementary data

Supplementary material Including Supplementary Tables 1 - 5.
