# Supplementary material for: Multidimensional expectations of radiotherapy among patients with cancer – A cross-sectional study (ExpectRT)
Source: Tech Innov Patient Support Radiat Oncol. 2026 Aug 4;39:100422. doi: 10.1016/j.tipsro.2026.100422 (PMC13485650; doi:10.1016/j.tipsro.2026.100422)
Supplement: Supplementary file 1 [file mmc1.pdf]

Multidimensional expectations of radiotherapy among patients with cancer – a cross-sectional study (ExpectRT)

Supplementary Table 1 Associations of Treatment Expectation Questionnaire (TEX-Q) mean values, Treatment benefit subscale values and Adverse events subscale values in the analyzed data set (n = 133) with ordinal covariables per Spearman correlation. p2

Supplementary Table 2 Associations of Treatment Expectation Questionnaire (TEX-Q) mean values, Treatment benefit subscale values, and Adverse events subscale values in the analyzed data set (n = 133) with continuous covariables per Pearson correlation. p2

Supplementary Table 3 Summary parameters and appreciation of assumptions of the "full" multiple regression models p3

Supplementary Table 4 Summary parameters and appreciation of assumptions of the "final" multiple regression models p3

Supplementary Table 5 Assessment of variable selection stability concerning the backwards elimination ("final") models via bootstrap inclusion frequencies. Bold font indicates selected variables p4

Supplementary Table 1 Associations of Treatment Expectation Questionnaire (TEX-Q) mean values, Treatment benefit subscale values and Adverse events subscale values in the analyzed data set (n = 133) with ordinal covariables per Spearman correlation. Abbreviations: CI, 95% – Confidence interval; PSCC, Patient Satisfaction with Cancer Care Questionnaire; SDM, shared decision-making

	**TEX-Q mean**				**TEX-Q Treatment benefit subscale**				**TEX-Q Adverse events subscale**			
**Variable**	ρ	Lower 95%-CI	Upper 95%-CI	p	ρ	Lower 95%-CI	Upper 95%-CI	p	ρ	Lower 95%-CI	Upper 95%-CI	p
Education	-0.25	-0.40	-0.08	**0.004**	-0.18	-0.34	-0.01	**0.043**	0.05	-0.12	0.22	0.5
Level of SDM [PSCC]	0.05	-0.12	0.22	0.5	0.10	-0.08	0.26	0.2	0.03	-0.14	2.0	0.7
Level of received needed advice [PSCC]	0.05	-0.12	0.22	0.5	0.05	-0.12	2.2	0.5	-0.11	-0.28	0.07	0.2

Supplementary Table 2 Associations of Treatment Expectation Questionnaire (TEX-Q) mean values, Treatment benefit subscale values, and Adverse events subscale values in the analyzed data set (n = 133) with continuous covariables per Pearson correlation. Abbreviations: CI, Confidence interval; QoL, Quality of Life

	**TEX-Q mean**				**TEX-Q Treatment benefit subscale**				**TEX-Q Adverse events subscale**			
**Variable**	r	Lower 95%-CI	Upper 95%-CI	p	r	Lower 95%-CI	Upper 95%-CI	p	r	Lower 95%-CI	Upper 95%-CI	p
Age	-0.02	-0.19	0.15	0.84	0.08	-0.09	0.25	0.33	0.07	-0.10	0.24	0.43
Distress	-0.11	-0.28	0.06	0.19	0.04	-0.14	0.20	0.68	0.25	0.08	0.40	**0.004**
Global health status/ QoL	0.07	-0.10	0.24	0.42	0.10	-0.07	0.27	0.25	-0.02	-0.19	0.15	0.85
Rated previous experience of radiotherapy [a]	0.26	-0.10	0.56	0.15	0.17	-0.20	0.49	0.37	-0.26	-0.56	0.10	0.15
Rated improvements after previous radiotherapy [a]	0.45	0.11	0.70	**0.01**	0.36	0.00	0.64	0.05	-0.20	-0.53	0.17	0.28

[a] Subgroup analysis of those patients who had previous radiotherapy (n=31)

Supplementary Table 3 Summary parameters and appreciation of assumptions of the "full" multiple regression models

Model Summary	TEX-Q mean	TEX-Q Treatment benefit subscale	TEX-Q Adverse events subscale
Number of observations	133	133	133
F(12, 120)	1.93	1.51	2.1
Prob > F	0.04	0.13	0.02
R-squared	0.16	0.13	0.18
Adj. R-squared	0.08	0.04	0.09
Durbin-Watson-Statistics	2.2	2.1	1.9
Highest VIF of independent variables		1.81	
Lowest tolerance of independent variables		0.55	
Assumption of homoscedasticity		Not violated per balanced distribution of residuals around baseline	
Assumption of normality and linearity		Not violated per Q-Q plot	
Variable entry method		Simultaneous entry	

Supplementary Table 4 Summary parameters and appreciation of assumptions of the "final" multiple regression models

Model Summary	TEX-Q mean	TEX-Q Treatment benefit subscale	TEX-Q Adverse events subscale
Number of observations	133	133	133
F statistics	F(3, 129) = 7.03	F(2, 130) = 6.69	F(4, 128) = 5.97
Prob > F	<.001	0.002	<.001
R-squared	0.14	0.09	0.16
Adj. R-squared	0.12	0.08	0.13
Durbin-Watson-Statistics	2.1	2.1	1.9
Highest VIF of independent variables	1.05	1.0	1.05
Lowest tolerance of independent variables	0.94	0.99	0.95
Assumption of homoscedasticity		Not violated per balanced distribution of residuals around baseline	
Assumption of normality and linearity		Not violated per Q-Q plot	
Variable entry method		Backward elimination (p-value thresholds: 0.05 for entry and 0.1 for removal)	

Supplementary Table 5 Assessment of variable selection stability concerning the backwards elimination ("final") models via bootstrap inclusion frequencies. Bold font indicates selected variables

	TEX-Q mean		TEX-Q Treatment benefit subscale		TEX-Q Adverse events subscale	
**Variable**	**Final model**	**Inclusion frequency**	**Final model**	**Inclusion frequency**	**Final model**	**Inclusion frequency**
Sex	No	30.6%	No	22.1%	No	22.2%
Age	No	22.5%	No	23.0%	No	25.5%
Cohabitation status	**Yes**	**81.5%**	No	20.3%	**Yes**	**89.1%**
Education	**Yes**	**98.4%**	**Yes**	**89.3%**	No	31.4%
Indication of radiotherapy	No	22.3%	**Yes**	**84.6%**	**Yes**	**60.3%**
Concomitant Chemotherapy	**Yes**	**48.4%**	No	40.9%	No	25.1%
Hospitalized	No	38.4%	No	34.0%	**Yes**	**61.1%**
Previous radiotherapy	No	14.2%	No	28.1%	No	14.2%
Level of SDM	No	25.0%	No	34.6%	No	30.0%
Level of needed advice received	No	27.5%	No	20.8%	No	34.6%
Distress	No	36.6%	No	43.1%	**Yes**	**88.9%**
Global health status/ QoL	No	30.9%	No	42.6%	No	37.2%
